# Age modifies the prognostic relevance of extracellular water-to-total body water ratio in rheumatoid arthritis: a retrospective cohort study

**DOI:** 10.1186/s41927-026-00668-2

**Published:** 2026-06-25

**Authors:** Daisuke Kobayashi, Yoko Wada, Masanori Sudo, Eriko Hasegawa, Shunsuke Sakai, Ayako Wakamatsu, Yukiko Nozawa, Hiroe Sato, Takeshi Kuroda, Suguru Yamamoto

**Affiliations:** 1 Division of Clinical Nephrology and Rheumatology, Kidney Research Center, Niigata University Graduate School of Medical and Dental Sciences, 1-757 Asahimachi-dori, Chuo-ku, Niigata, 951-8510 Japan; 2 Department of Clinical Rheumatology, Niigata Rinko Hospital, Niigata, Japan; 3 Niigata Rheumatic Center, Shibata, Japan; 4 Health Administration Center, Niigata University, Niigata, Japan

**Keywords:** Rheumatoid arthritis, ECW/TBW, Frailty, Physiological reserve, Ageing, Mortality

## Abstract

**Background:**

Clinicians often recognise broader deterioration in a patient’s overall condition that is not fully captured by conventional disease-specific measures. Ageing is associated with reduced physiological reserve and altered body fluid distribution. However, objective markers that capture these changes remain limited. Because the extracellular water-to-total body water ratio (ECW/TBW) reflects fluid distribution, we hypothesised that its prognostic relevance is age-dependent rather than uniform.

**Methods:**

In this retrospective cohort study, 186 adults with rheumatoid arthritis underwent bioelectrical impedance analysis in 2017 and were followed until December 2025. The primary outcome was a composite of all-cause death or transition to do-not-attempt-resuscitation/best supportive care (DNAR/BSC) status. Cox proportional hazards models including an age × ECW/TBW interaction term were used. Age-stratified analyses used the cohort median age (68 years).

**Results:**

During a median follow-up of 95.2 months, 17 participants experienced death or transition to DNAR/BSC status. Age was strongly associated with risk (HR per 10-year increase 2.53, 95% CI 1.47–4.34; *p* < 0.001). In age-adjusted models, ECW/TBW was not independently associated with the outcome (HR per 0.01 increase 1.10, 95% CI 0.88–1.37; *p* = 0.40). In contrast, the age × ECW/TBW interaction was significant (HR 1.22, 95% CI 1.05–1.42; *p* = 0.010), suggesting that the association between ECW/TBW and the outcome differed according to age. Elevated ECW/TBW was associated with higher risk among older participants (HR 3.10, 95% CI 1.21–8.63; *p* = 0.018), but not among younger individuals. In competing-risk analyses, elevated ECW/TBW was associated with a higher cumulative incidence of non-planned admissions among older participants (Gray’s test *p* = 0.014).

**Conclusions:**

The prognostic relevance of ECW/TBW appeared to vary by age, emerging primarily in later life rather than functioning as a uniform risk marker. These exploratory findings suggest that ECW/TBW may reflect aspects of age-related physiological vulnerability and may help identify older individuals at increased risk of non-planned hospitalisations and other unstable clinical trajectories. As a simple non-invasive measure, ECW/TBW may complement disease activity-centred assessment, although external validation is required.

**Supplementary Information:**

The online version contains supplementary material available at https://doi.org/10.1186/s41927-026-00668-2.

## Background

Ageing is characterised by a progressive decline in physiological reserve and resilience, resulting in a reduced capacity to maintain homeostasis under stress [1]. This process is accompanied by alterations in body composition and fluid distribution that extend beyond overt disease activity.

Rheumatoid arthritis (RA) represents a clinically relevant model of chronic inflammatory ageing. As a systemic inflammatory disease associated with functional decline and increased mortality, RA reflects the cumulative biological burden of long-standing inflammation [2]. Although disease-modifying therapies have improved inflammatory control, patients with RA remain vulnerable to adverse outcomes in later life. Mortality risk in RA reflects not only inflammatory burden but also ageing-related factors such as frailty and sarcopenia [3–5], highlighting the limitations of prognostication based solely on disease activity.

Frailty has emerged as a key determinant of outcomes in rheumatic diseases and is more prevalent in RA than in other arthritides [3, 5]. Reduced muscle mass and sarcopenia are associated with increased mortality risk [4]. Frailty encompasses both quantitative muscle loss and qualitative alterations in tissue composition and fluid distribution, reflecting diminished physiological reserve. However, existing frailty indices do not fully capture progressive, systemic decline, and clinically meaningful vulnerability often remains difficult to quantify in routine practice.

Bioelectrical impedance analysis provides a non-invasive method for assessing whole-body composition and fluid distribution [6]. The extracellular water-to-total body water ratio (ECW/TBW) reflects relative extracellular expansion and intracellular water reduction, patterns observed in advanced ageing and chronic disease [7]. Elevated ECW/TBW has been associated with mortality across diverse clinical settings, including community-dwelling populations [8], cancer with sarcopenia [9], cardiovascular surgery [10], chronic heart failure [11], and haemodialysis populations [12, 13]. However, the reported prognostic value of ECW/TBW has not been entirely consistent, and it remains unclear whether its clinical significance is uniform across patient groups or instead depends on underlying physiological context.

Cachexia and frailty overlap and jointly contribute to adverse outcomes, reflecting a multidimensional decline in physiological reserve [14, 15]. In clinical practice, however, prognosis in RA is often framed primarily in terms of inflammatory activity and comorbidity burden. Yet clinicians frequently recognise broader vulnerability that is not fully captured by these conventional measures. Disturbances that are well tolerated in younger individuals may carry fundamentally different clinical implications when compensatory reserve is reduced. This observation raises the possibility that the impact of physiological markers such as ECW/TBW may not be uniform but modified by the level of underlying vulnerability, particularly age.

We therefore hypothesised that the prognostic value of ECW/TBW is not uniform but age-dependent, emerging primarily with advancing age. To test this hypothesis, we examined whether baseline ECW/TBW is associated with long-term risk of death or transition to do-not-attempt-resuscitation/best supportive care (DNAR/BSC) status in individuals with RA, and specifically evaluated whether this association is modified by age.

## Methods

### Study design and participants

This retrospective cohort study was conducted at the rheumatology outpatient department of a tertiary care university hospital in Niigata, Japan. In 2017, adult outpatients with RA attending outpatient clinics were included if they underwent bioelectrical impedance analysis as part of a prospectively planned body composition assessment. Eligible patients were aged ≥ 18 years, and baseline body composition data were available for all included participants.

Baseline bioimpedance measurements were obtained between April and June 2017 within a prospectively designed clinical study approved by the Niigata University Ethics Review Committee for Research Involving Human Subjects (approval number: 2016-0046), which was independent of the present research question.

The current analysis represents a retrospective secondary analysis of these prospectively collected data, for which separate ethical approval was obtained (approval number: 2022 − 0282).

Clinical outcomes were identified through retrospective review of electronic medical records up to December 2025. Follow-up time was defined from the date of baseline assessment to the occurrence of the outcome event or the end of the available records, whichever came first.

RA was diagnosed by the treating rheumatologists according to standard classification criteria used in routine clinical practice [16, 17].

### Body composition assessment

Measurements were performed using a validated multi-frequency bioelectrical impedance device (InBody S20, Biospace Co., Ltd., Seoul, Korea) according to the manufacturer’s standard protocols, with participants in the supine position. All measurements were obtained at baseline only, and longitudinal changes were not assessed.

ECW/TBW was analysed as a continuous variable and additionally categorised into quartiles, with the highest quartile defined as elevated ECW/TBW to enhance clinical interpretability. Given the limited number of outcome events, this categorisation was also used to support model stability.

### Analytical framework

Based on our hypothesis that the prognostic value of ECW/TBW may vary with context, we examined whether its association with outcomes differed by age. To do this, we included an interaction term between ECW/TBW and age in multivariable models.

Age was treated as a continuous variable to preserve statistical power and avoid arbitrary categorisation. We additionally performed age-stratified analyses as descriptive approaches to facilitate interpretation.

### Clinical variables

Functional status was assessed at baseline using the modified Health Assessment Questionnaire (mHAQ), a simplified derivative of the Health Assessment Questionnaire (HAQ), which has been validated in Japanese populations and is widely used in patients with rheumatoid arthritis [18, 19].

Detailed longitudinal data on treatment exposure and nutritional status were not available, and residual confounding cannot be excluded. Given the limited number of outcome events, the primary model was kept parsimonious. Additional covariates were examined individually in separate models.

In sensitivity analyses, additional covariates including CDAI, albumin, appendicular skeletal muscle mass index (ASMI), body mass index (BMI), estimated glomerular filtration rate (eGFR), prednisolone (PSL) dose, RA duration, history of heart failure, and modified Health Assessment Questionnaire Disability Index (mHAQ-DI) were incorporated individually into separate models.

Accordingly, we focused on a parsimonious model including age and ECW/TBW, with particular attention to their interaction, and performed sensitivity analyses to examine the consistency of the findings.

Given the limited number of outcome events, all multivariable and interaction analyses were considered exploratory. Additional covariates were incorporated individually into separate models to examine the consistency of the age × ECW/TBW interaction, rather than to establish a fully adjusted causal model. Missing covariate data were handled by complete-case analysis for each model, and no imputation was performed.

### Outcomes

The primary outcome was a composite of all-cause mortality and transition to DNAR/BSC status during follow-up. To improve reproducibility and reduce outcome misclassification, transition to DNAR/BSC status was counted only when the medical record explicitly documented a DNAR order, transition to best supportive care, or both, in the context of advanced clinical deterioration [20]. Cases with ambiguous descriptions of clinical deterioration, supportive care, or end-of-life discussions without explicit DNAR/BSC documentation were excluded from the composite outcome classification.

DNAR/BSC transition was not considered equivalent to death or a surrogate for mortality. Rather, it was treated as a distinct clinically documented milestone reflecting advanced deterioration and a shift in goals of care. The timing of DNAR/BSC decisions may be influenced by clinical judgement, patient and family preferences, institutional practice, and healthcare-system factors; therefore, this endpoint requires cautious interpretation. In this cohort, death and DNAR/BSC events were mutually exclusive. ECW/TBW measurements were not available to treating clinicians at the time of DNAR/BSC decision-making and were not used to guide clinical management in routine practice.

### Statistical analysis

Continuous variables are presented as the mean ± SD or median (interquartile range [IQR]), and categorical variables as n (%). Kaplan–Meier methods were used with log-rank tests for group comparisons.

Cox proportional hazards models were used to estimate hazard ratios (HRs) with 95% confidence intervals (CIs). Age was analysed per 10-year increase and ECW/TBW per 0.01 increase in all Cox models. To improve interpretability of regression coefficients in interaction models, both variables were rescaled to clinically meaningful units and mean-centred prior to analysis. An interaction term between age and ECW/TBW was included to assess effect modification. Because the magnitude of interaction terms depends on variable scaling, the interaction effect was interpreted alongside age-stratified analyses to aid clinical interpretation.

In addition, age-stratified analyses based on the cohort median age (68 years) were performed as descriptive analyses to visualise differential risk patterns. Alternative age cut-offs were also examined to assess the consistency of the findings.

Because of the small number of death events, death-only sensitivity analyses were conducted using Firth-penalised Cox regression to reduce small-sample bias [21]. A two-sided p-value < 0.05 was considered statistically significant. All analyses were performed using R (version 4.3.1). Sample size varied across models because of missing covariate data.

Further sensitivity analyses, including models with alternative age cut-offs, analyses treating ECW/TBW as a continuous variable in younger patients, and exploratory Fine–Gray models for non-planned admission, were performed to examine the consistency of the findings [22, 23]. Details are provided in the Supplementary Methods and Supplementary Tables S2–S6.

### Competing-risk analysis of clinical trajectories

To further characterise the nature of the observed age-dependent risk and to explore differences in patterns of clinical deterioration, we performed competing-risk analyses focusing on hospital admission types.

Cumulative incidence functions (CIFs) were estimated, treating planned and non-planned hospital admissions as competing events. Planned admissions were defined as scheduled hospitalisations for elective procedures or disease management, whereas non-planned admissions were defined as urgent or emergency hospitalisations.

For the competing-risk analysis, only the first hospital admission after baseline was classified as planned or non-planned, and the two admission types were treated as competing first events.

We focused on the first hospital admission after baseline to maintain a mutually exclusive first-event competing-risk framework and to avoid the additional modelling assumptions required for recurrent admissions. These analyses were exploratory and intended to support clinical interpretation of possible trajectory patterns, rather than to provide definitive evidence regarding the relationship between ECW/TBW and admission type.

### Ethics

This study was conducted in accordance with the Declaration of Helsinki and was approved by the Niigata University Ethics Review Committee for Research Involving Human Subjects (approval number: 2022 − 0282). The requirement for informed consent was waived by the Niigata University Ethics Review Committee for Research Involving Human Subjects because of the retrospective nature of the study and the use of de-identified clinical data. Institutional approval was obtained prior to data collection.

This study was reported in accordance with the STROBE statement [24].

### Use of artificial intelligence tools

During manuscript preparation, ChatGPT (OpenAI) was used to assist with English language editing and phrasing suggestions. The authors reviewed and approved all content and take full responsibility for the final manuscript.

## Results

### Study population

A total of 186 patients with RA were included in the study (of whom 141 [75.8%] were female). The median baseline age was 68 years (IQR 59–75). The median ECW/TBW was 0.399 (IQR, 0.389–0.406), and the upper quartile corresponded to ECW/TBW > 0.406. The median follow-up duration was 95.2 months (IQR, 52.7–102.0). Baseline characteristics according to the composite outcome are shown in Table 1, and those stratified by age group are summarised in Supplementary Table S1. The distribution of age and ECW/TBW supported the evaluation of potential effect modification by age in subsequent analyses.

**Table 1:** Baseline characteristics according to composite outcome status

Variable	Overall (*n* = 186)	No composite outcome (*n* = 169)	Death or DNAR/BSC transition (*n* = 17)
Age, years	68 [59–75]	68 [59–75]	73 [69–81]
Female sex, n (%)	141 (75.8%)	129 (76.3%)	12 (70.6%)
RA duration, years	12.0 [5.2–19.0]	12.0 [5.0–19.0]	14.0 [11.0–19.0]
CDAI	8.1 [4.0–12.1]	8.2 [3.6–12.1]	7.7 [5.7–10.7]
mHAQ-DI	0.1 [0.0–0.5]	0.1 [0.0–0.5]	0.1 [0.0–0.6]
ECW/TBW	0.399 [0.389–0.406]	0.398 [0.389–0.405]	0.410 [0.403–0.419]
BMI, kg/m²	21.7 [19.6–23.4]	21.8 [19.7–23.5]	21.3 [17.6–22.2]
ASMI, kg/m²	4.7 [4.1–5.7]	4.8 [4.2–5.7]	4.2 [3.9–4.6]
Albumin, g/dL	4.0 [3.7–4.2]	4.0 [3.7–4.2]	3.8 [3.5–3.9]
eGFR, mL/min/1.73 m²	74.5 [59.8–91.6]	75.4 [60.1–92.2]	72.0 [54.2–84.1]
PSL dose, mg/day	2.0 [0.0–5.0]	2.0 [0.0–5.0]	4.0 [0.0–6.0]
History of heart failure, n (%)	6 (3.2%)	6 (3.6%)	0 (0.0%)
Follow-up duration, months	95.1 [52.7–101.9]	98.3 [62.8–102.1]	54.3 [39.5–68.4]

Continuous variables are presented as median [IQR], and categorical variables as n (%)

ECW/TBW, extracellular water-to-total body water ratio; CDAI, Clinical Disease Activity Index; mHAQ-DI, modified Health Assessment Questionnaire Disability Index; ASMI, appendicular skeletal muscle mass index; eGFR, estimated glomerular filtration rate; PSL, prednisolone; DNAR, do-not-attempt-resuscitation; BSC, best supportive care

### Primary analysis: age-dependent association (interaction)

During follow-up, 17 patients experienced death or transition to DNAR/BSC status. Among the 17 composite outcome events, 11 were deaths and 6 were transitions to DNAR/BSC status, with no overlapping cases. In age-adjusted analyses without an interaction term, age was the strongest predictor of the composite outcome (HR per 10-year increase 2.53, 95% CI 1.47–4.34; *p* < 0.001). ECW/TBW was not independently associated with the outcome after adjustment for age (HR per 0.01 increase 1.10, 95% CI 0.88–1.37; *p* = 0.40).

However, in Cox models including an age × ECW/TBW interaction term, with age scaled per 10-year increase and ECW/TBW per 0.01 increase, a statistically significant interaction was observed (HR 1.22, 95% CI 1.05–1.42; *p* = 0.010), suggesting that the association between ECW/TBW and the outcome differed according to age (Table 2; Supplementary Table S3).

The age × ECW/TBW interaction showed directionally consistent results across exploratory multivariable models adjusting individually for CDAI, albumin, ASMI, eGFR, BMI, PSL dose, RA duration, history of heart failure, and mHAQ-DI, although overlap with functional status and residual confounding cannot be excluded (Table 3).

**Table 2:** Cox model for the age × ECW/TBW interaction

Variable	HR (95% CI)	*p*-value
Age (per 10-year increase)	2.54 (1.36–4.74)	0.003
ECW/TBW (per 0.01 increase)	1.10 (0.87–1.40)	0.419
Sex (male vs. female)	2.56 (0.82–7.99)	0.106
**Age × ECW/TBW interaction**	**1.22 (1.05–1.42)**	**0.010**

Hazard ratios (HRs) were estimated using a Cox proportional hazards model including age (per 10-year increase), ECW/TBW (per 0.01 increase), their interaction term, and sex. Age and ECW/TBW were mean-centred prior to analysis. In models including an interaction term, the main effects represent conditional effects at the centering values of the interacting variable and should not be interpreted in isolation

**Table 3:** Exploratory consistency analyses of the age × ECW/TBW interaction across multivariable Cox models

Model	Additional covariate	*N*	Events	HR (95% CI)	*p*-value
Base model	None	186	17	1.22 (1.05–1.42)	0.010
Model 1	CDAI	186	17	1.22 (1.05–1.42)	0.010
Model 2	Albumin	184	16	1.20 (1.03–1.40)	0.018
Model 3	ASMI	186	17	1.22 (1.05–1.42)	0.011
Model 4	eGFR	186	17	1.23 (1.05–1.42)	0.008
Model 5	BMI	186	17	1.22 (1.05–1.41)	0.009
Model 6	PSL dose	186	17	1.23 (1.04–1.45)	0.014
Model 7	RA duration	186	17	1.24 (1.06–1.44)	0.006
Model 8	History of heart failure	186	17	1.21 (1.05–1.41)	0.010
Model 9	mHAQ-DI	186	17	1.21 (1.04–1.42)	0.016

Hazard ratios (HRs) represent the effect of the interaction term between age (per 10-year increase) and ECW/TBW (per 0.01 increase), estimated from separate Cox proportional hazards models. Each model was adjusted for sex and one additional covariate listed in the table. Age and ECW/TBW were mean-centred prior to analysis. Additional covariates were entered in their original scales. Sample size varied slightly across models due to missing data

### Age-stratified Kaplan–Meier analyses

Kaplan–Meier analyses stratified by age were performed to further examine the observed interaction between age and ECW/TBW. In younger patients (< 68 years), ECW/TBW did not discriminate event-free survival for the composite outcome of death or DNAR/BSC transition (log-rank *p* = 0.53). In older patients (≥ 68 years), however, high ECW/TBW (> 0.406) was associated with a significantly higher risk compared with lower ECW/TBW (quartiles 1–3) (log-rank *p* = 0.0052).

**Fig. 1:**
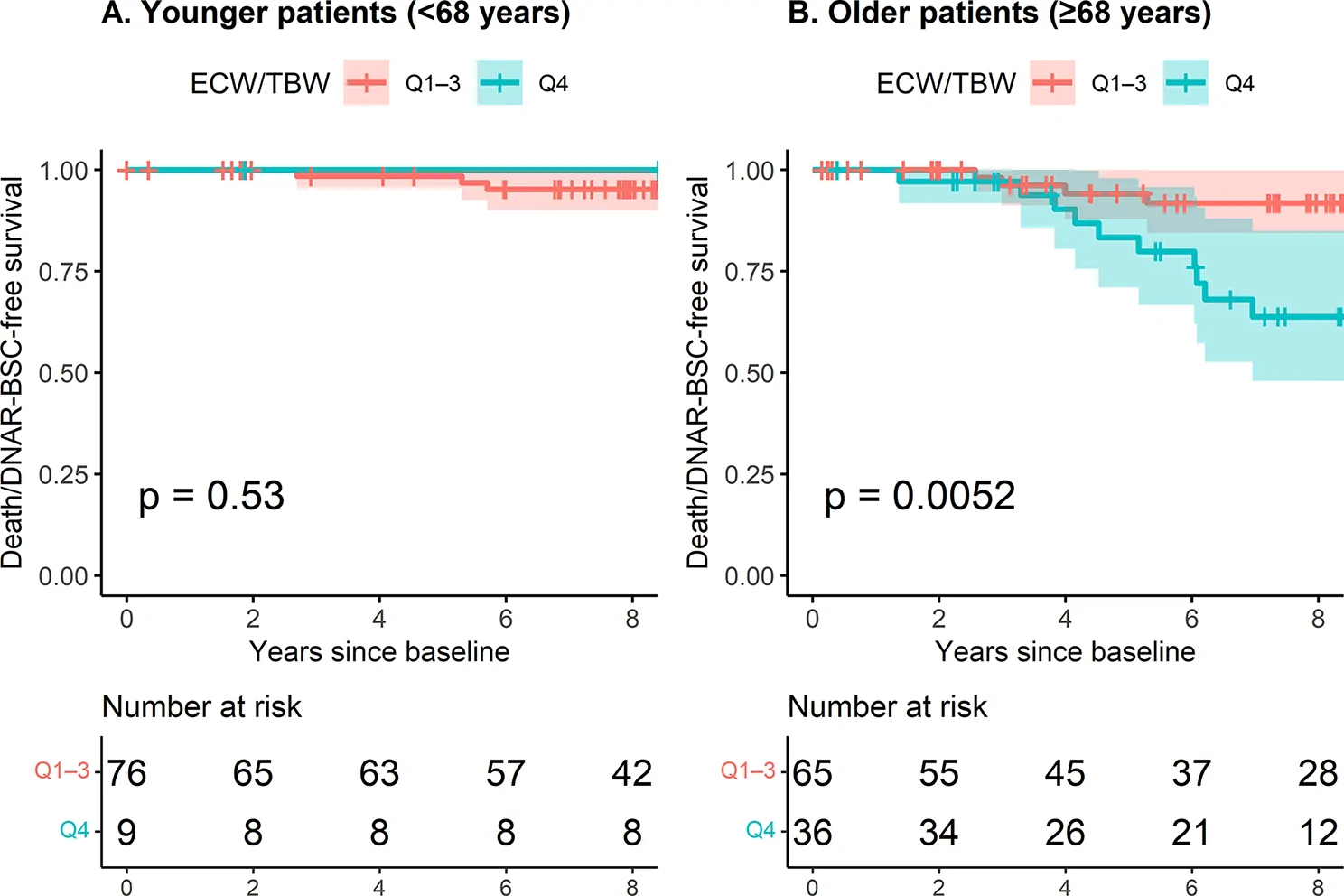
Age-stratified risk according to ECW/TBW. Kaplan–Meier curves stratified by extracellular water-to-total body water ratio (ECW/TBW) in (**A**) younger (< 68 years) and (**B**) older (≥ 68 years) patients. Within each age group, ECW/TBW was categorised as high (top quartile > 0.406) or low (quartiles 1–3). In younger patients, ECW/TBW did not discriminate the composite outcome of death or transition to DNAR/BSC status (log-rank *p* = 0.53), whereas in older patients, high ECW/TBW was associated with an increased risk of the composite outcome (log-rank *p* = 0.0052). Shaded areas represent 95% confidence intervals, and numbers at risk are shown below each panel. Absolute event counts were 0/9 in the high ECW/TBW group and 3/76 in the low ECW/TBW group among younger patients, and 10/36 and 4/65 among older patients. These event counts highlight the limited number of outcomes, particularly in younger participants, and support cautious interpretation of subgroup analyses

In the older group, events occurred in 10 of 36 patients with high ECW/TBW and in 4 of 65 patients with lower ECW/TBW (Fig. 1). In age-stratified Cox regression analyses using Firth correction, high ECW/TBW (Q4 vs. Q1–3) was associated with increased risk among older patients (HR 3.10, 95% CI 1.21–8.63; *p* = 0.018). In contrast, among younger patients, descriptive Cox analysis treating ECW/TBW as a continuous variable yielded statistically unstable estimates because only three events occurred (Supplementary Table S2).

### Competing-risk analysis of clinical trajectories

To further characterise the observed age-dependent association, cumulative incidence function (CIF) analyses were performed to examine differences in clinical trajectories according to hospital admission type.

**Fig. 2:**
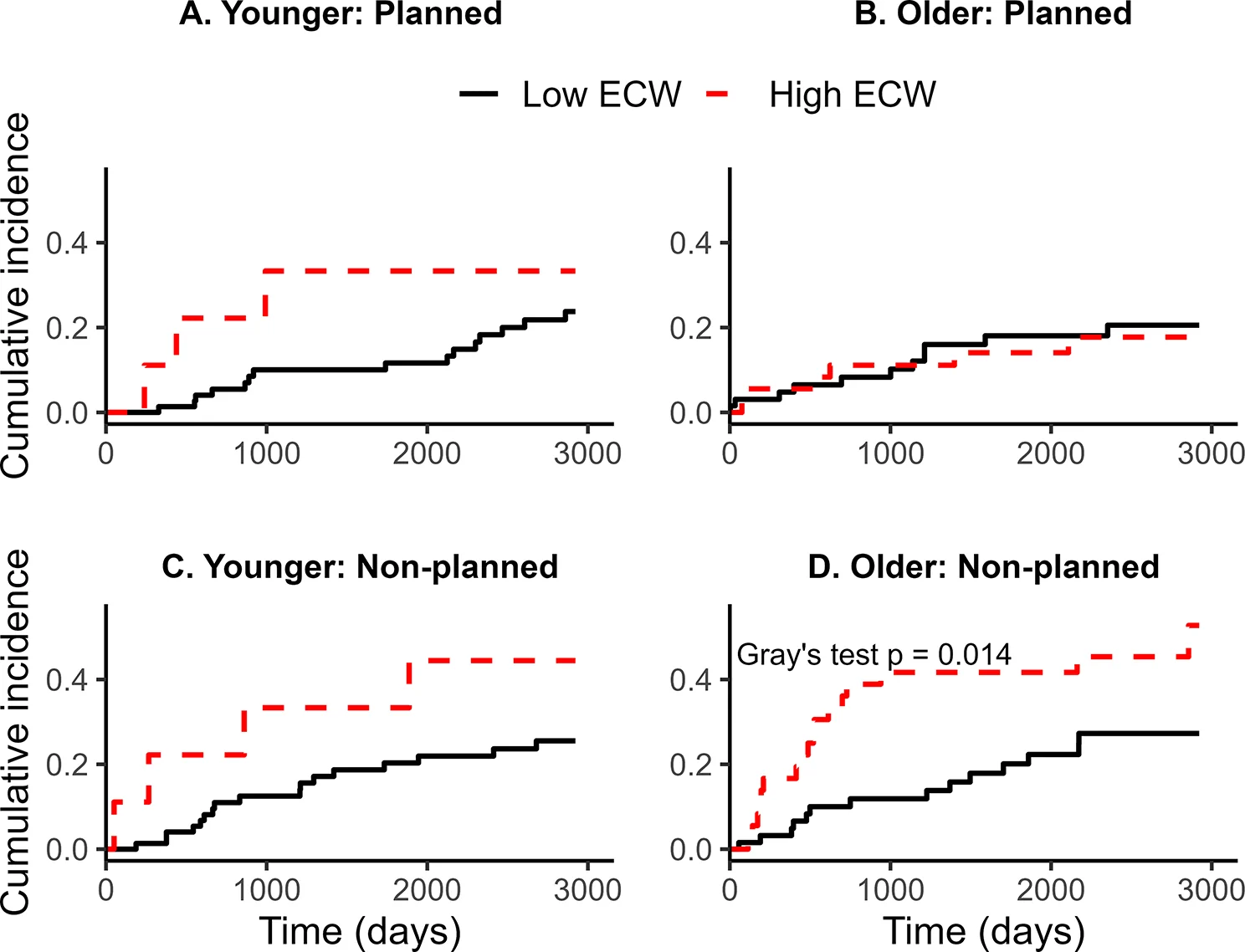
Cumulative incidence of planned and non-planned admissions. Cumulative incidence functions (CIFs) accounting for competing risks are shown for planned admissions (top panels) and non-planned admissions (bottom panels), stratified by age group: younger (< 68 years; left panels) and older (≥ 68 years; right panels). Within each panel, patients were categorised into high ECW/TBW (top quartile; red dashed line) and low ECW/TBW (quartiles 1–3; black solid line). In younger patients, high ECW/TBW showed numerically higher cumulative incidence of both planned and non-planned admissions. In contrast, in older patients, ECW/TBW demonstrated different patterns according to admission type, with a higher incidence of non-planned admissions in the high ECW/TBW group, without a clear difference in planned admissions. Among older participants, the cumulative incidence of non-planned admissions was higher in the high ECW/TBW group (Gray’s test *p* = 0.014), whereas no significant difference was observed for planned admissions (*p* = 0.78)

Among older participants, cumulative incidence differed according to ECW/TBW group. While no significant difference was observed for planned admissions (Gray’s test *p* = 0.78), the incidence of non-planned admissions was significantly higher in the high ECW/TBW group (*p* = 0.014) (Fig. 2). An exploratory Fine-Gray model showed a directionally consistent association between high ECW/TBW and non-planned admission (Supplementary Table S4).

### Sensitivity analyses

#### Death-only sensitivity analysis

In a separate death-only sensitivity analysis using Firth-penalised Cox regression, the age × ECW/TBW interaction remained directionally consistent and statistically significant, although estimates were imprecise because only 11 deaths occurred (HR 1.18, 95% CI 1.04–1.34; *p* = 0.039; Supplementary Table S5). This analysis was performed to address the distinction between all-cause mortality and DNAR/BSC transition.

Analyses using alternative age cut-offs (65, 70, and 75 years) yielded consistent results, although estimates were imprecise because of small subgroup event numbers (Supplementary Table S6; Supplementary Figure S2).

Sex-adjusted survival curves corresponding to the age-stratified analyses are presented in Supplementary Figure S1.

## Discussion

In this cohort of individuals with long-standing RA, the prognostic relevance of ECW/TBW appeared to vary by age, with risk separation becoming more evident primarily in older individuals. This age-dependent pattern was consistent with the observed age × ECW/TBW interaction in the primary model. Although chronological age remained the dominant determinant of risk, ECW/TBW may provide additional prognostic information in older patients with RA.

A key finding was the discrepancy between main-effect and interaction-based analyses. ECW/TBW was not independently associated with the outcome in models without an interaction term, whereas explicitly modelling effect modification revealed a significant association confined to older individuals. This indicates that the prognostic meaning of ECW/TBW is context-dependent rather than uniform across populations.

This pattern is compatible with the interpretation that ECW/TBW may reflect aspects of physiological vulnerability, although direct measures of frailty, biological ageing, and longitudinal body composition were not available. ECW/TBW likely overlaps with frailty, nutritional status, sarcopenia, inflammation, comorbidity, and functional decline, and should not be interpreted as an independent biological construct. Rather, it may serve as a pragmatic, non-invasive marker that captures overlapping dimensions of vulnerability in older patients with RA.

Frailty is increasingly recognised as a key determinant of outcomes in rheumatic diseases, including RA [25, 26]. Our previous work has shown that biological disease-modifying antirheumatic drugs can influence nutritional status and muscle-related parameters in RA [27]. Consistent with this, body composition–related measures such as phase angle have been associated with adverse outcomes in older populations [28, 29].

Building on these observations, the present study extends this perspective to later-stage vulnerability and prognosis. The findings are also consistent with reports across diverse clinical settings [8–13] linking elevated ECW/TBW to adverse outcomes, supporting a broader interpretation of RA as a model of chronic inflammatory ageing in which cumulative inflammatory burden and ageing interact to erode biological resilience.

From a clinical perspective, ECW/TBW may serve as a simple, non-invasive marker to identify older patients at risk of unstable clinical trajectories, particularly those involving non-planned hospitalisations, complementing conventional disease activity–centred assessments.

Several limitations should be acknowledged. ECW/TBW was assessed at a single time point, and longitudinal changes were not evaluated; therefore, we could not determine whether elevated ECW/TBW reflected persistent vulnerability or a transient physiological state. Residual confounding remains substantial because direct frailty assessments, detailed comorbidity burden, prior hospitalisation history, socioeconomic factors, longitudinal treatment exposure, and longitudinal inflammatory or nutritional status were unavailable. The number of outcome events was limited, and this was a single-centre study, which may restrict generalisability. However, the uniformity of clinical practice, outcome definitions, and measurement protocols likely reduced heterogeneity and strengthened internal validity. The ECW/TBW cutoff used in this study was cohort-derived and should not be interpreted as a universal clinical threshold; external validation is needed to determine clinically applicable cutoff values. The composite outcome, which included transition to DNAR/BSC status, requires careful interpretation, as such decisions may be influenced by clinical judgement, patient and family preferences, institutional practice, and healthcare-system factors. Because of the limited number of events, even parsimonious models including interaction terms may be susceptible to overfitting; therefore, effect estimates and p-values should be interpreted as exploratory rather than definitive. The competing-risk analyses of planned and non-planned admissions were exploratory and intended only to support clinical interpretation of possible trajectory patterns, not to provide definitive evidence.

Because this was a single-centre retrospective secondary analysis of observational data, the present findings should be interpreted as associations for prognostic stratification rather than evidence of biological causality. ECW/TBW may be a marker that co-occurs with age-related vulnerability, frailty, comorbidity, or nutritional decline, but the present design cannot determine whether altered fluid distribution directly contributes to adverse outcomes.

In this study, DNAR/BSC transition was not treated as a surrogate for mortality, but as a clinically meaningful milestone reflecting advanced deterioration and a shift in goals of care. Its inclusion allowed assessment of clinically important late-stage deterioration beyond death alone; nevertheless, death-only analyses were performed separately as sensitivity analyses.

This composite outcome may capture clinically important deterioration beyond disease activity alone, but it should not be interpreted as a direct biological endpoint. In clinical practice, clinicians may recognise changes in patient trajectories that are not fully explained by inflammatory burden or conventional disease-specific measures. Our findings raise the possibility that ECW/TBW may help identify older patients with such unstable clinical trajectories, including non-planned hospitalisations or changes in goals of care. Further studies are needed to determine whether incorporating ECW/TBW into clinical assessment can improve risk stratification or treatment planning.

These findings should be considered hypothesis-generating and require validation in independent cohorts.

## Conclusions

In conclusion, the prognostic relevance of ECW/TBW in RA appeared to vary by age, becoming more evident in later life. These exploratory findings suggest that ECW/TBW may reflect aspects of physiological vulnerability that become increasingly relevant as physiological reserve declines with ageing. ECW/TBW may help identify older individuals at risk of unstable clinical trajectories, including non-planned hospitalisations, but the findings should be considered hypothesis-generating and require external validation before clinical application.

## Electronic Supplementary Material

Below is the link to the electronic supplementary material.

Supplementary Material 1

Supplementary Material 2

Supplementary Material 3

## Data Availability

The datasets generated and/or analysed during the current study are not publicly available due to the protection of individual privacy but are available from the corresponding author on reasonable request, subject to institutional approval.

## Abbreviations

ASMI: Appendicular skeletal muscle mass index
BMI: Body mass index
BSC: Best supportive care
CDAI: Clinical Disease Activity Index
CIF: Cumulative incidence function
CI: Confidence interval
DNAR: Do-not-attempt-resuscitation
ECW/TBW: Extracellular water-to-total body water ratio
eGFR: Estimated glomerular filtration rate
HR: Hazard ratio
IQR: Interquartile range
mHAQ-DI: Modified Health Assessment Questionnaire Disability Index
PSL: Prednisolone
RA: Rheumatoid arthritis
SD: Standard deviation
