# Supplementary material for: Age modifies the prognostic relevance of extracellular water-to-total body water ratio in rheumatoid arthritis: a retrospective cohort study
Source: BMC Rheumatol. 2026 Jun 25;10:68. doi: 10.1186/s41927-026-00668-2 (PMC13563699; doi:10.1186/s41927-026-00668-2)
Supplement: Supplementary file 1 [file 41927_2026_668_MOESM1_ESM.docx]

**Supplementary Methods**

**Analysis in younger patients**

Among patients younger than 68 years, the number of composite outcome events was limited (n = 3). A Cox proportional hazards model treating ECW/TBW as a continuous variable was therefore performed for descriptive purposes only. Given the small number of events, hazard ratio estimates were considered statistically unstable, and no causal or protective interpretation was intended.

**Age-stratified and sensitivity analyses**

To explore potential age dependency in the association between ECW/TBW and composite outcome of death or transition to DNAR/BSC status, we performed prespecified age-stratified analyses using a cutoff of 68 years, corresponding to the median age of the cohort. In addition, sensitivity analyses were conducted using alternative age cutoffs (≥65, ≥70, and ≥75 years) to examine the consistency and directionality of risk separation associated with high ECW/TBW (Q4 vs Q1–3). For these analyses, Cox proportional hazards models were fitted separately for each age threshold. The same definition of the composite outcome of death or transition to DNAR/BSC status was used across models, although the number of patients and events differed according to each age threshold.

**Statistical considerations**

No multiplicity adjustment was applied, as sensitivity analyses were conducted to support interpretation of the primary analysis and were not intended to generate independent inferential claims.

**Exploratory Fine-Gray analysis for non-planned admission**
As an exploratory sensitivity analysis, Fine-Gray subdistribution hazard models were used to examine non-planned hospital admission as an alternative outcome, treating planned admission as a competing event. Elevated ECW/TBW was defined as the highest quartile (Q4) of the cohort distribution, consistent with the categorisation used in cumulative incidence analyses. Age was analysed per 10-year increase and included as a continuous variable. Models were adjusted for sex. Given the exploratory nature of this analysis, results were interpreted cautiously.

**Graphical presentation**

Sex-adjusted survival curves stratified by age group were generated to visually compare event-free survival according to ECW/TBW category (Supplementary Figure S1). For visual comparison of effect estimates across alternative age cutoffs, hazard ratios for high ECW/TBW (Q4 vs Q1–3) were displayed using a forest plot (Supplementary Figure S2). This graphical summary was intended to illustrate the directionality and consistency of risk separation rather than to compare statistical significance across models.

**Supplementary Table S1. Baseline characteristics by age group**

| **Variable** | **Overall (N=186)** | **Younger (N =85)**  **(<68 years)** | **Older (N =101)**  **(≥68 years)** | **P value** |
| --- | --- | --- | --- | --- |
| Age, years | 68 [59–75] | 59 [52–62] | 75 [70–79] | <0.001 |
| Female, n (%) | 141 (75.8%) | 63 (74.1%) | 78 (77.2%) | 0.748 |
| Height, cm | 156 [151–163] | 159 [156–166] | 152 [148–161] | <0.001 |
| Body weight, kg | 53 [46–61] | 56 [49–64] | 50 [45–57] | <0.001 |
| BCM, kg | 20.0 [16.3–23.7] | 20.8 [18.5–26.6] | 18.5 [16.2–22.1] | <0.001 |
| BCM/H², kg/m² | 8.10 [7.16–9.21] | 8.40 [7.28–9.87] | 7.73 [7.14–8.73] | 0.016 |
| BCM/BW, % | 37.83 [32.77–43.02] | 38.83 [32.75–44.12] | 37.50 [33.90–41.28] | 0.404 |
| ECW/TBW | 0.40 [0.39–0.41] | 0.40 [0.38–0.40] | 0.40 [0.40–0.41] | <0.001 |
| ECW/TBW Q4 (>0.406), n (%) | 45 (24.2%) | 9 (10.6%) | 36 (35.6%) | <0.001 |
| Follow-up duration, months | 95.1 [52.7–101.9] | 100.7 [82.1–102.3] | 86.5 [44.4–101.4] | 0.009 |

Footnote

*Values are presented as median [interquartile range] or number (%).
ECW/TBW Q4 was defined as the highest quartile of ECW/TBW in the overall cohort (cutoff = 0.406).
P values were calculated using the Mann–Whitney U test for continuous variables and the χ² test or Fisher’s exact test for categorical variables, as appropriate.*

**Supplementary Table S2. Cox proportional hazards analysis of ECW/TBW as a continuous variable in younger patients (<68 years)**

| **Variable** | **HR (per 0.01 increase)** | **95% CI** | **p value** |
| --- | --- | --- | --- |
| ECW/TBW | 0.73 | 0.55–0.95 | 0.021 |

Footnote

*Given the limited number of events (n = 3), estimates should be interpreted descriptively and not as evidence of a protective association. The model was adjusted for sex. Results were similar in models without sex adjustment.*

**Supplementary Table S3. Sensitivity analyses of the interaction between age and ECW/TBW**

| **Variable** | **HR (95% CI)** | **p-value** |
| --- | --- | --- |
| Age × ECW/TBW interaction (continuous) | 1.22 (1.05–1.42) | 0.010 |
| Age × ECW/TBW interaction (age dichotomised at 68 years) | 1.78 (1.29–2.46) | <0.0001 |
| Age × ECW/TBW interaction (Firth penalised Cox model) | 1.18 (1.01–1.33) | 0.0389 |

Footnote

*Hazard ratios (HRs) represent the interaction effect between age and ECW/TBW. In the continuous model, both variables were treated as continuous. In the dichotomised model, age was categorised at 68 years. A Firth penalised Cox model was applied to reduce small-sample bias due to the limited number of events. All models were adjusted for sex. Results were consistent in direction across modelling approaches. For the Firth penalised Cox model, 95% confidence intervals and p-values were based on profile likelihood.*

**Supplementary Table S4. Fine-Gray analysis of non-planned hospital admission using elevated ECW/TBW (highest quartile)**

| Variable | sHR (95% CI) | p-value |
| --- | --- | --- |
| Age (per 10-year increase) | 1.20 (0.89–1.62) | 0.23 |
| High ECW/TBW (Q4 vs Q1–3) | 2.47 (1.24–4.91) | 0.0098 |
| Sex (male vs female) | 2.14 (1.12–4.08) | 0.021 |

*Subdistribution hazard ratios (sHRs) were estimated using Fine-Gray competing risk models, with non-planned hospital admission treated as the event of interest and planned admission as a competing event. The model included age (per 10-year increase), sex, and ECW/TBW. Elevated ECW/TBW was defined as the highest quartile (Q4) of the cohort distribution. This categorisation was consistent with the cumulative incidence analyses and was used to facilitate comparison across analytical approaches. This exploratory analysis was performed to complement the cumulative incidence findings and to assess whether the association between elevated ECW/TBW and non-planned admission remained directionally consistent after accounting for the competing risk of planned admissions.* **Supplementary Table S5. Death-only analysis using Firth-penalised Cox regression**

| **Variable** | **HR (95% CI)** | **p-value** |
| --- | --- | --- |
| Age (per 10-year increase) | 3.17 (1.37–7.34) | 0.002 |
| ECW/TBW (per 0.01 increase) | 0.87 (0.69–1.10) | 0.287 |
| Sex (male vs female) | 2.19 (0.54–8.85) | 0.271 |
| **Age × ECW/TBW interaction** | **1.18 (1.04–1.34)** | **0.039** |

*Footnote*

*Hazard ratios (HRs) were estimated using Firth-penalised Cox regression because of the limited number of death events. Age was mean-centred and scaled per 10-year increase, and ECW/TBW was mean-centred and scaled per 0.01 increase.*

**Supplementary Table S6. Sensitivity analyses in older subgroups defined by alternative age thresholds**

| **Age cut** | **Variable** | **HR** | **95% CI** | **p value** | **N / Event** |
| --- | --- | --- | --- | --- | --- |
| ≥65 years | ECW/TBW Q4 vs Q1–3 | 3.55 | 1.33–10.26 | 0.012 | 116 / 16 |
| ≥70 years | ECW/TBW Q4 vs Q1–3 | 4.36 | 1.34–17.91 | 0.013 | 81 / 12 |
| ≥75 years | ECW/TBW Q4 vs Q1–3 | 13.99 | 1.65–1824.83 | 0.011 | 51 / 8 |

*Footnote*

*Cox proportional hazards models were fitted separately in subgroups defined by age thresholds of ≥65, ≥70, and ≥75 years, with ECW/TBW (Q4 vs Q1–3) as the exposure of interest. All models were adjusted for sex. Associations were directionally consistent across alternative age thresholds, although estimates became less precise at higher thresholds because of the smaller sample size and fewer events.*

**Supplementary Figure S1. Age-stratified, sex-adjusted survival curves according to extracellular water-to-total body water ratio (ECW/TBW).**

*Sex-adjusted survival curves for the composite outcome of death or transition to do-not-attempt-resuscitation/best supportive care (DNAR/BSC) status are shown according to ECW/TBW category in younger (<68 years; panel A) and older (≥68 years; panel B) patients. ECW/TBW was categorised into high (Q4) and low (Q1–3) groups based on the upper quartile threshold of the overall cohort. Curves were estimated from Cox proportional hazards models adjusted for sex using the conditional method. P-values shown in each panel were derived from likelihood ratio tests comparing sex-adjusted Cox models with and without ECW/TBW category.*

**Supplementary Figure S2.**

Sensitivity analysis of age cut–specific hazard ratios for high ECW/TBW

*Forest plot showing hazard ratios for death or transition to do-not-attempt-resuscitation/best supportive care (DNAR/BSC) status associated with high extracellular water–to–total body water ratio (ECW/TBW; Q4 vs Q1–3) across alternative age cutoffs (≥65, ≥70, and ≥75 years).
Points indicate hazard ratios and horizontal lines represent 95% confidence intervals. The vertical dashed line denotes a hazard ratio of 1.0.
Hazard ratios were consistently above 1.0 across all cutoffs and increased with higher age thresholds (≥65 years: HR 3.55 [95% CI 1.33–10.26]; ≥70 years: HR 4.36 [95% CI 1.34–17.91]; ≥75 years: HR 13.99 [95% CI 1.65–1824.83]), although confidence intervals widened at higher thresholds because of smaller sample sizes and fewer events. All models were adjusted for sex. These findings were consistent with the primary age-stratified analysis.*
